# A comparative analysis of cardiopulmonary features in patients with systemic sclerosis and mixed connective tissue disease: results from SOPHIE registry

**DOI:** 10.3389/fmed.2025.1626546

**Published:** 2025-08-13

**Authors:** Mi Zhou, Bing-hua Wang, Ming-jiao Liao, Rui Yang, Jing Tan, Wen-jun Zhang, Chun-ka Wong, Chung-Wah Siu, Lixue Yin

**Affiliations:** ^1^Department of Ultrasound in Medicine, Sichuan Provincial People's Hospital Wenjiang Hospital, Chengdu, China; ^2^Cardiology Division, Department of Medicine, Queen Mary Hospital, The University of Hong Kong, Hong Kong, Hong Kong SAR, China; ^3^Ultrasound in Cardiac Electrophysiology and Biomechanics Key Laboratory of Sichuan Province, Sichuan Provincial People's Hospital, University of Electronic Science and Technology of China, Chengdu, China

**Keywords:** pulmonary function, cardiac function, left ventricular ejection fraction, echocardiography, electrocardiography

## Abstract

**Purpose:**

We aimed to identify various cardiopulmonary involvement patterns in patients with systemic sclerosis (SSc) and mixed connective tissue disease (MCTD).

**Methods:**

Laboratory experiments, pulmonary function test, 6-min walk distance (6MWD), transthoracic echocardiography, and 12-lead electrocardiography were used to evaluate cardiopulmonary function in patients with SSc and those with MCTD..

**Results:**

A total of 138 patients with SSc and 56 patients with MCTD were enrolled in the study. Patients in the MCTD group exhibited a higher systolic blood pressure (SBP) (128.73 ± 16.82 vs. 121.95 ± 21.22, *p* = 0.03), diastolic blood pressure (DBP) (75.84 ± 10.96 vs. 70.79 ± 14.47, *p* = 0.02), and mean arterial pressure (MAP) (92.89 ± 13.02 vs. 87.17 ± 13.81, *p* = 0.009) compared to those in the SSc group. Levels of SaO_2_ at pre-6MWD (96.55 ± 2.61 vs. 98.67 ± 2.05, *p* < 0.001) and post-6MWD (95.73 ± 5.46 vs. 98.40 ± 2.73, *p* = 0.002) were markedly lower in MCTD patients compared to SSc patients. Laboratory analysis indicated that MCTD patients had lower platelet (PLT) counts (221.78 ± 71.88 vs. 253.96 ± 80.13, *p* = 0.01) and higher troponin T (TNT) (31.32 ± 74.85 vs.12.83 ± 16.30, *p* = 0.04) and brain natriuretic peptide (BNP) levels (193.35 ± 351.59 vs. 57.37 ± 53.68, p = 0.04) compared to patients with SSc. Pulmonary function tests revealed a decreased FEF50% predicted value (88.69 ± 44.58 vs. 122.86 ± 59.57, *p* < 0.001) and a higher proportion of patients with FEF75% predicted value <65% (28.26% vs. 10.71%, *p* = 0.009) in the SSc group compared to the MCTD group. Compared to SSc patients, patients with MCTD showed an increased left ventricular end-diastolic volume (LVEDV) (91.85 ± 32.87 vs. 73.32 ± 24.75, *p* < 0.001) and left ventricular end-systolic volume (LVESV) (30.59 ± 16.13 vs. 24.10 ± 8.99, *p* = 0.006), alongside decreased LVEF measured by the Simpson method (62.48 ± 6.33 vs. 66.58 ± 6.94, *p* < 0.001). Additionally, a higher proportion of patients in the MCTD group demonstrated a moderate or higher probability of pulmonary hypertension (PH) (39.29% vs. 13.04%, *p* = 0.000).

**Conclusion:**

In this cross-sectional analysis, we found small airway dysfunction in patients with SSc and impaired left ventricular systolic function in patients with MCTD. These findings indicate that there is significant heterogeneity in the cardiopulmonary involvement patterns, although these patterns are both connective tissue diseases with similar disease duration and comorbidity burden.

## Introduction

Systemic sclerosis (SSc) is a rare multisystem connective tissue disease (CTD) characterized by progressive fibrosis of the skin and visceral organs, predominantly involving the lung, cardiovascular system, kidney, and gastrointestinal tract, as well as immune-induced microvascular dysfunction (1). Mixed connective tissue disease (MCTD) is a distinct clinical entity identified by the simultaneous or successive manifestations of different CTDs, including SSc, polymyositis/dermatomyositis, rheumatoid arthritis (RA), and systemic lupus erythematosus (SLE) (2).

CTDs associated with pulmonary artery hypertension (PAH) and comorbid interstitial lung disease (ILD) exhibited a poorer 1-year survival rate compared to patients with idiopathic PAH (3). Although previous studies have identified SSc-associated PAH (SSc-PAH) as the predominant group of CTD-associated PAH (CTD-PAH), accounting for approximately 74% of cases, studies from the Asia region report that SLE and primary Sjögren’s syndrome are the most prevalent etiologies in CTD-PAH (4, 5). In addition, the prevalence of ILD linked with SSc and MCTD has been reported to vary from 33.4 to 85.3% and from 0.7 to 67%, respectively, due to heterogeneity in the diagnostic techniques and criteria across different countries (6–9). In fact, PAH associated with SSc is frequently secondary to interstitial fibrosis, whereas, in patients with MCTD, it is usually driven by intimal hyperplasia and intima-media hypertrophy of the pulmonary arteries.

A limited number of studies have investigated the cardiac and lung function between SSc and MCTD. Valentini et al. compared the prevalence of mitral peak early-to-late diastolic filling velocity (E/A) ratio <1 and a diffusing capacity of carbon monoxide (DLCO) < 80% predicted value between SSc and MCTD patients in the early stage, indicating that there were no significant differences between the two groups. However, cardiopulmonary characteristics in SSc and MCTD populations were not fully studied due to the occult nature of the disease (10). The primary objective of the study was to systematically assess the cardiopulmonary involvement variation between SSc and MCTD through the integrated application of 6-min walk distance (6MWD), transthoracic echocardiography, electrocardiography, and pulmonary function testing, to promote the development of targeted disease management strategies.

## Materials and methods

### Study design

This cross-sectional multicenter study was conducted between 2018 and 2023 among patients who registered in the CTD cohort in Hong Kong SAR and China. The inclusion criteria of this study were as follows: (1) age ≥ 18 years, (2) satisfying the classification criteria of SSc (11) or diagnostic criteria of MCTD (12), and (3) willingness to provide written informed consent to participate in the study. The study design and detailed registry information were previously published (13). The study protocol was registered in ClinicalTrials.gov (NCT03446339) and has been approved by the Institutional Review Board (UW-16-2076) of the University of Hong Kong, Sichuan Provincial People’s Hospital (SPPH), and SPPH-Wenjiang Hospital (ER-2023-014). All study practices adhered to the Declaration of Helsinki and principles of ethics in medical research.

### Clinical data collection

Clinical and demographic data, including age, age of onset, disease duration, gender, body mass index (BMI), blood pressure, and medical history, were collected from electronic patient records at the time of enrollment in this study. Laboratory results, including the levels of hemoglobin (Hb), white blood cell (WBC) counts, platelet counts (PLT), levels of urea and creatinine, troponin T (TNT), brain natriuretic peptide (BNP), as well as the presence of autoantibodies, were recorded. The enzyme-linked immunosorbent assay (ELISA) method was used to determine and count anti-Scl 70, anti-centromere, and anti-ribonucleoprotein (anti-RNP) antibodies.

### Six-minute walk distance (6MWD)

The 6MWD practical procedure was performed by a skilled cardiology nurse following the standardized protocol outlined in the American Thoracic Society guidelines (14). In short, 6MWD evaluates the maximum distance a patient can walk on flat and solid ground within 6 min. The oxygen saturation (SaO_2_) of pre- and post-6MWD was systematically measured to assess the patient’s cardiopulmonary function and exercise capacity during the test.

### Pulmonary function test (PFT)

Lung volume parameters, including the predicted value of forced vital capacity (FVC), forced expiratory volume in the first second (FEV1), forced expiratory flow at 25% (FEF25), 50% (FEF50) and 75% (FEF75), total lung capacity (TLC), residual volume (RV), RV/TLC ratio, and DLCO divided by alveolar volume (DLCO/VA), were acquired during the test. All measurements of PFT were recorded as a percentage (%) of the predicted value.

### Conventional echocardiography

Transthoracic echocardiography (TTE) was performed according to the clinical guidelines released by the American Society of Echocardiography (15). Left cardiac cavity measurements include the minor (LA_minor) and major (LA_major) dimensions of the left atrium, area of the left atrium (LAA), dimensions of the interventricular septum at end-systole (IVSs) and end-diastole (IVSd), and dimensions of the left ventricular posterior wall at end-systole (LVPWs), end-diastole (LVPWd), left ventricular end-systolic (LVEDV), and end-diastolic volume (LVESV). The left ventricular ejection fraction was calculated using the Teichholz method (LVEF_m mode) and biplane Simpson method (LVEF_simpson), respectively. LV diastolic function was evaluated by the peak early (E) and late (A) mitral diastolic velocity, E/A ratio, E deceleration time (EDT), and isovolumetric relaxation time (IVRT). Right cardiac cavity measurements include the minor (RA_minor) and major (RA_major) dimensions of the right atrium, the area of the right atrium (RAA), the basal (RV_basal), middle (RV_mid), and longitudinal (RV_long) dimensions of the right ventricle, the proximal (RV_proximal) and distal (RV_distal) dimensions of the right ventricular outflow tract, and the right ventricular end-systolic (RVESA) and end-diastolic (RVEDA) area. Right ventricular systolic function was evaluated by fractional area change (RV_FAC). The diagnostic criterion for pulmonary hypertension (PH) is resting mean pulmonary arterial pressure ≥25 mmHg, as determined by right heart catheterization. The probability of PH in the study was assessed by transthoracic echocardiography, based on peak tricuspid regurgitant velocity to estimate right ventricular systolic pressure combined with other “PH” echocardiographic signs based on the guidelines for the diagnosis of PH (16).

### Electrocardiography

The 12-lead electrocardiography (ECG) was performed for all participants. The incidence of atrial fibrillation (AF), premature atrial complex (PAC), premature ventricular complex (PVC), sinus tachycardia, sinus bradycardia, and right bundle branch block (RBBB) was recorded with numbers and percentages during the examination. The diagnostic criteria for right ventricular hypertrophy (RVH) were described in previous research (17, 18). The ECG practice and results interpretation were carried out by two independent investigators.

### Statistical analysis

Normally distributed continuous variables (CVs) were described as mean ± standard deviation (SD). Qualitative variables were expressed as numbers and percentages. Comparisons of clinical and demographic features, blood sample results, PFT detection, TTE measurements, and ECG investigation between the two groups were performed using an independent samples t-test if CVs satisfied with independence and normal distribution. In qualitative variables, chi-square or Fisher’s exact test was used to calculate the column proportions and differences between the two groups. A *p*-value of <0.05 was considered a statistically significant difference in all tested hypotheses. IBM SPSS version 29.0.1.0 and GraphPad Prism version 10.1.1 were used for data analysis and figure drafting.

## Results

### Clinical and demographic characteristics

**Figure 1:**
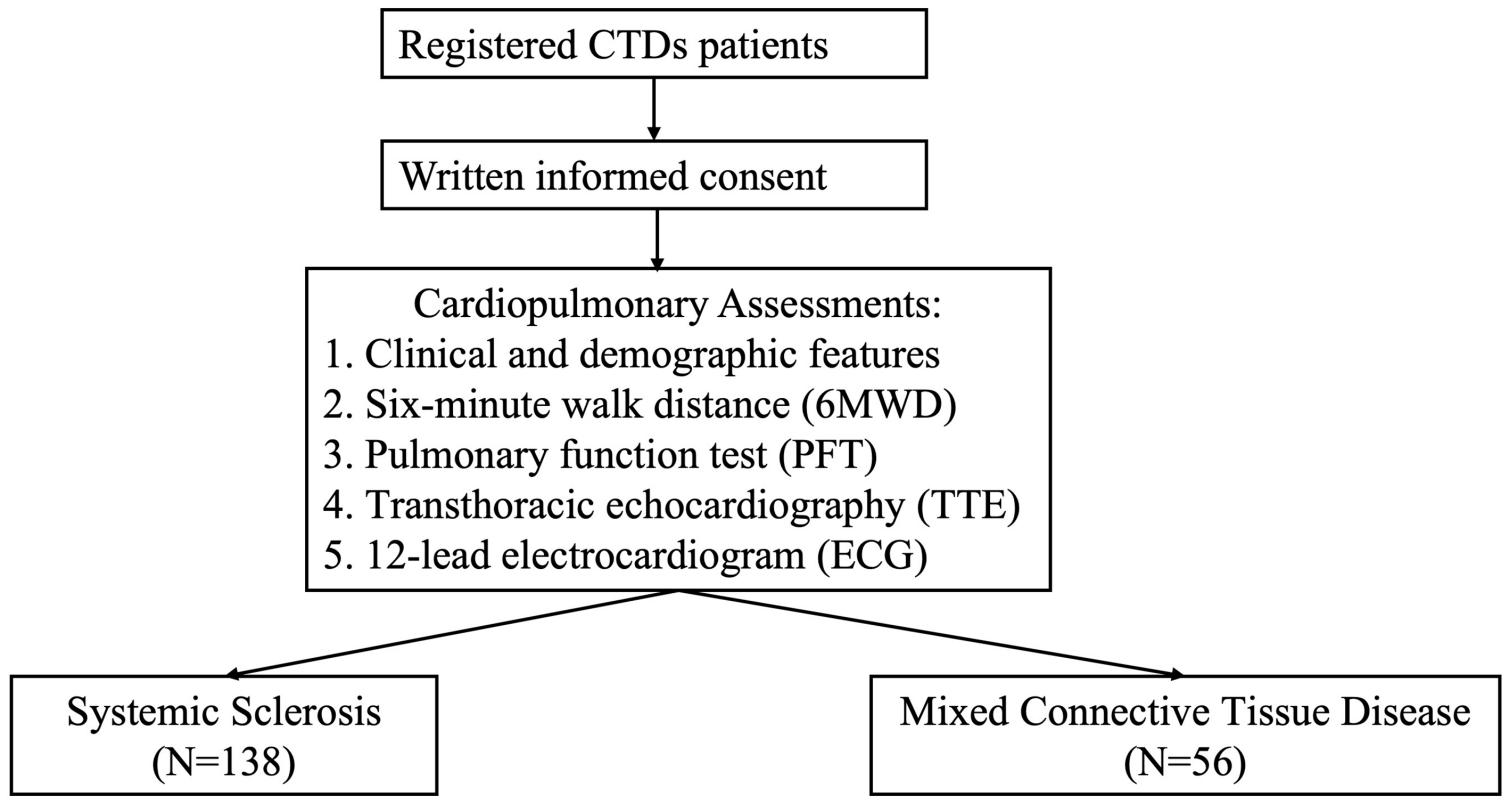
The flow chart of the study.

A total of 138 patients diagnosed with SSc and 56 patients with MCTD were included from the CTD-registered cohort for the present study analysis (Figure 1). The difference in enrollment between SSc and MCTD patients was mainly because the incidence of SSc is significantly higher than that of MCTD. The combined prevalence of SSc was 17.6 per 100,000 persons (19), while the prevalence of MCTD was 3.8 ~ 6.4 per 100,000 persons (20). Approximately 85.7% of the patients had developed other CTD conditions before the diagnosis of MCTD, including 15 with scleroderma, 7 with dermatomyositis, 8 with SLE, 10 with polymyositis, and 8 with RA. Age, age of onset, disease duration, gender, and BMI were comparable between the two groups (Table 1). Patients with MCTD exhibited higher SBP (128.73 ± 16.82 vs. 121.95 ± 21.22, *p* = 0.03), DBP (75.84 ± 10.96 vs. 70.79 ± 14.47, *p* = 0.02), and MAP (92.89 ± 13.02 vs. 87.17 ± 13.81, *p* = 0.009) compared to the SSc group. Raynaud phenomenon (RP) was more prevalent in the SSc group than in the MCTD group (88.41% vs. 57.14%; *p* < 0.000). No significant differences were tested in comorbidities, including hypertension (HT), diabetes mellitus (DM), coronary artery disease (CAD), or chronic kidney disease (CKD), between the two groups. Additionally, levels of SaO_2_ at pre-6MWD (96.55 ± 2.61 vs. 98.67 ± 2.05, *p* < 0.001) and post-6MWD (95.73 ± 5.46 vs. 98.40 ± 2.73, *p* = 0.002) were markedly lower in MCTD patients compared to those with SSc.

**Table 1:** Comparison of demographic and clinical characteristics between SSc and MCTD patients.

Characteristics	SSc (*N* = 138)	MCTD (*N* = 56)	*p-*value
Sociodemographic characteristics			
Age, years	57.46 ± 14.95	55.47 ± 12.88	0.39
Age of onset, years	45.59 ± 17.51	44.31 ± 14.90	0.66
Disease duration, years	11.47 ± 11.00	10.58 ± 10.57	0.63
Gender: man, *n* (%)	12(8.7)	6(10.70)	0.41
BMI	21.87 ± 3.88	22.20 ± 3.59	0.58
Smoking status, *n* (%)	14 (10.14)	4 (7.14)	0.60
Test performed			
SBP, mmHg	121.95 ± 21.22	128.73 ± 16.82	0.03
DBP, mmHg	70.79 ± 14.47	75.84 ± 10.96	0.02
MAP, mmHg	87.17 ± 13.81	92.89 ± 13.02	0.009
HR, bpm	76.91 ± 12.01	80.66 ± 13.09	0.06
6MWD, m	426.23 ± 93.14	455.54 ± 96.48	0.06
Sao2_pre6MWT, %	98.67 ± 2.05	96.55 ± 2.61	<0.001
Sao2 Post6MWT, %	98.40 ± 2.73	95.73 ± 5.46	0.002
Rheumatological features			
RP, *n* (%)	122 (88.41)	32 (57.14)	<0.000
Telangiectasia, *n* (%)	39 (28.26)	12 (21.43)	0.37
ILD, *n* (%)	24 (17.39)	12 (21.43)	0.54
Comorbidity			
HT, *n* (%)	22 (15.94)	14 (25.00)	0.16
DM, *n* (%)	8 (5.80)	2 (3.57)	0.73
CAD, *n* (%)	2 (1.45)	2 (3.57)	0.58
CKD, *n* (%)	10 (7.25)	2(3.57)	0.51

BMI, body mass index; SBP, systolic blood pressure; DBP, diastolic blood pressure; MAP, mean arterial pressure; HR, heart rate; RP, Raynaud phenomenon; ILD, interstitial lung disease; HT, hypertension; DM, diabetes mellitus; CAD, coronary artery disease; CKD, chronic kidney disease; 6MWD, 6-min walk distance; SaO_2_, arterial oxygen saturation.

### Laboratory findings

Comparisons of laboratory data between patients with SSc and MCTD are summarized in Table 2. There were no significant differences in the levels of Hb, WBC, urea, and creatinine between the two groups. However, MCTD patients demonstrated significantly lower PLT counts (221.78 ± 71.88 vs. 253.96 ± 80.13, *p* = 0.01) and higher TNT and BNP levels (*p* = 0.04) compared to patients with SSc. Additionally, anti-Scl-70 and anti-centromere antibody positivity rates were higher in SSc patients (anti-Scl-70: *p* < 0.000; anti-centromere: *p* = 0.03), whereas anti-RNP positivity was more prevalent in MCTD patients (42.86% vs. 12.32%, *p* < 0.000).

**Table 2:** Comparison of laboratory findings between SSc and MCTD patients.

Parameters	SSc (*N* = 138)	MCTD (*N* = 56)	*p-*value
Hb, g/dL	12.53 ± 1.62	12.45 ± 1.23	0.76
WBC, 10^9^/L	6.28 ± 2.03	7.16 ± 5.67	0.27
PLT, 10^9^/L	253.96 ± 80.13	221.78 ± 71.88	0.01
PLT < 100 × 10^3^/mL	1 (0.72)	3 (5.36)	0.07
Urea, μmol/L	5.25 ± 2.59	5.02 ± 1.59	0.53
Creatinine, μmol/L	68.44 ± 46.92 (68.35)	63.43 ± 17.68 (63.19)	0.44
TNT, ng/L	12.83 ± 16.30 (16.69)	31.32 ± 74.85 (23.45)	0.04
BNP, pg./mL	57.37 ± 53.68 (79.82)	193.35 ± 351.59 (131.13)	0.04
Antibodies			
Anti-Scl-70, *n* (%)	63 (45.65)	4 (7.14)	<0.000
Anti-centromere, *n* (%)	38 (27.54)	7 (12.50)	0.03
Anti-RNP, *n* (%)	17 (12.32)	24 (42.86)	<0.000

Hb, hemoglobin; WBC, white blood count; PLT, platelet; TNT, troponin t; BNP, brain natriuretic peptide; Anti-RNP, anti-ribonucleoprotein.

### Pulmonary function in SSc and MCTD

**Figure 2:**
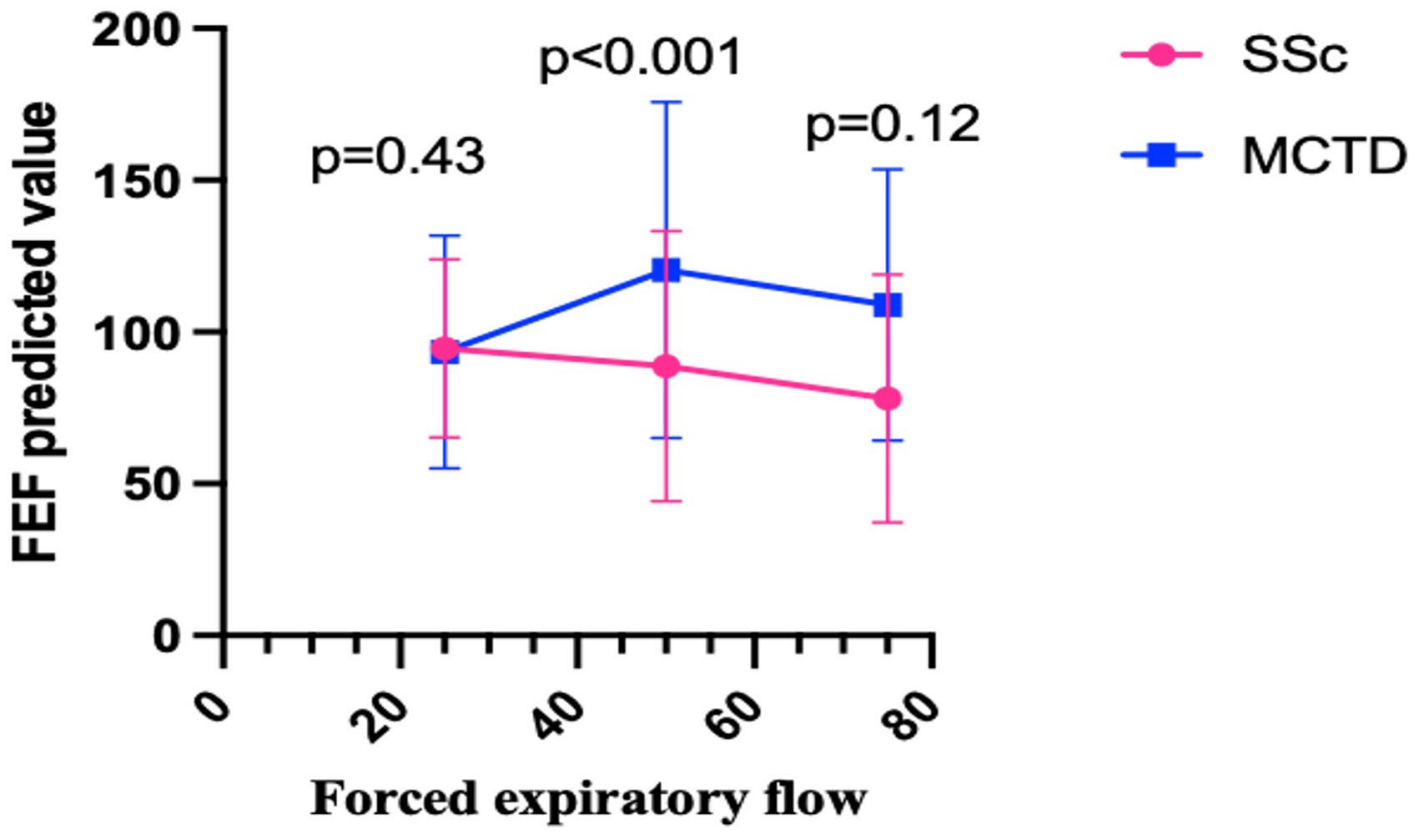
The serial alterations in FEF from 25% to 75% among the two groups.

The comparative analysis of PFT between the two groups revealed significant differences across specific parameters (Table 3). Compared to the MCTD group, patients in the SSc group exhibited a prominent reduction in FEF50 predicted value (88.69 ± 44.58 vs. 122.86 ± 59.57, *p* ≤ 0.001) and a higher proportion of patients had FEF75 < 65% (28.26% vs. 10.71%, *p* = 0.009). Additionally, an increased RV predicted value was observed among patients in the SSc group compared to those in the MCTD group (97.75 ± 38.61 vs. 85.82 ± 22.13, *p* < 0.028). The serial alterations in FEF from 25 to 75% among the two groups are shown in Figure 2.

**Table 3:** Comparison of pulmonary function between SSc and MCTD patients.

Parameters	SSc (*N* = 138)	MCTD (*N* = 56)	*p-*value
Test time to diagnosis, years	9.37 ± 10.97	8.29 ± 10.59	0.62
FVC, %	86.79 ± 22.57	83.92 ± 16.64	0.21
FEV1, %	87.89 ± 20.89	84.84 ± 19.52	0.20
FEV1/FVC ≥ 0.7 & FVC < 80%, *n* (%)	41(29.71)	15(26.79)	0.73
FEF25, %	94.50 ± 29.37	93.43 ± 38.33	0.43
FEF25 < 65%, *n* (%)	16 (11.59)	9 (16.07)	0.48
FEF50, %	88.69 ± 44.58	122.86 ± 59.57	<0.001
FEF50 < 65%, *n* (%)	24 (17.39)	7 (12.50)	0.52
FEF75, %	78.01 ± 40.88	68.70 ± 30.17	0.12
FEF75 < 65%, *n* (%)	39 (28.26)	6 (10.71)	0.009
TLC, %	91.46 ± 20.28	84.14 ± 16.28	<0.046
RV, %	97.75 ± 38.61	85.82 ± 22.13	<0.028
RV/TLC, %	103.99 ± 30.55	102.97 ± 12.43	0.84
DLCO/VA, %	85.09 ± 17.67	83.27 ± 17.78	0.59
DLCO<55%, *n* (%)	5 (3.62)	2 (3.57)	0.99
FVC/DLCO>1, *n* (%)	7 (5.07)	7 (12.50)	0.12

FVC, forced vital capacity; FEV1, forced expiratory volume in the first second; FEF25, forced expiratory flow at 25%; FEF50, forced expiratory flow at 50%; FEF75, forced expiratory flow at 75%; TLC, total lung capacity; RV, residual volume; DLCO, diffusing capacity of carbon monoxide; DLCO/VA, diffusing capacity of carbon monoxide divided by alveolar volume.

### Echocardiographic characteristics in SSc and MCTD

Echocardiographic assessments are summarized in Table 4. Compared to patients in the SSc group, patients in the MCTD group showed increased left ventricular dimension at end-diastole (LVEDD, 4.23 ± 0.50 vs. 4.41 ± 0.66, *p* = 0.04), LVEDV (91.85 ± 32.87 vs. 73.32 ± 24.75, *p* < 0.001), and LVESV (30.59 ± 16.13 vs. 24.10 ± 8.99, *p* = 0.006), alongside decreased LVEF_simpson (62.48 ± 6.33 vs. 66.58 ± 6.94, *p* < 0.001). However, no significant differences were tested in other measurements of the left atrium and left ventricle. Patients in the MCTD group demonstrated a greater RV_long dimension (*p* = 0.01) and a higher proportion of moderate or high probability of PH compared to patients in the SSc group (*p* = 0.000).

**Table 4:** Comparison of echocardiographic measurements between SSc and MCTD patients.

Echocardiographic measurements	SSc (*N* = 138)	MCTD (*N* = 56)	*P-*value
Left cardiac			
LA_minor, cm	3.79 ± 0.63	3.66 ± 0.72	0.24
LA_major, cm	4.83 ± 0.63	4.95 ± 0.72	0.26
LA_area, cm^2^	16.02 ± 3.54	15.80 ± 3.63	0.69
IVSd, cm	0.88 ± 0.17	0.84 ± 0.15	0.08
LVEDD, cm	4.23 ± 0.50	4.41 ± 0.66	0.04
LVPWd, cm	0.88 ± 0.16	0.84 ± 0.15	0.15
IVSs, cm	1.23 ± 0.21	1.21 ± 0.19	0.48
LVESD, cm	2.62 ± 0.36	2.76 ± 0.53	0.08
LVPWs, cm	1.31 ± 0.25	1.29 ± 0.22	0.60
LVEF_m mode, %	68.33 ± 4.95	68.37 ± 5.47	0.14
LVEDV, mL	73.32 ± 24.75	91.85 ± 32.87	<0.001
LVESV, mL	24.10 ± 8.99	30.59 ± 16.13	0.006
LVEF_Simpson, %	66.58 ± 6.94	62.48 ± 6.33	<0.001
LVEF<50%, *n* (%)	1(0.72)	2(3.57)	0.20
E, m/s	0.82 ± 0.18	0.81 ± 0.16	0.77
A, m/s	0.75 ± 0.22	0.72 ± 0.19	0.38
E/A ratio	1.16 ± 0.42	1.29 ± 0.89	0.18
EDT, ms	189.34 ± 48.54	205.64 ± 66.23	0.10
IVRT, ms	87.64 ± 19.24	83.37 ± 19.67	0.18
LVOT, cm	1.89 ± 0.19	2.00 ± 0.27	0.002
VTI _LVOT_, cm	21.99 ± 4.60	21.45 ± 4.65	0.47
CO, L/min	4.40 ± 1.33	5.18 ± 2.26	0.003
SVR, Woods	1704.93 ± 492.34	1626.78 ± 516.23	0.33
SV, mL	61.26 ± 16.56	68.48 ± 28.94	0.09
Right cardiac			
RA_minor, cm	3.22 ± 0.56	3.32 ± 0.61	0.24
RA_major, cm	4.38 ± 0.55	4.55 ± 0.65	0.06
RA area, cm^2^	12.80 ± 3.25	13.54 ± 3.41	0.16
RV_basal, cm	2.95 ± 0.41	2.93 ± 0.52	0.79
RV_mid, cm	2.68 ± 0.52	2.51 ± 0.58	0.057
RV_long, cm	5.35 ± 0.88	5.72 ± 0.94	0.01
RVOT proximal (cm)	2.64 ± 0.40	2.62 ± 0.39	0.73
RVOT_distal, cm	2.01 ± 0.34	2.11 ± 0.39	0.07
RVEDA, cm^2^	12.64 ± 4.11	12.66 ± 4.60	0.98
RVESA, cm^2^	6.67 ± 2.78	6.83 ± 2.71	0.69
RV_FAC, %	50.87 ± 7.08	49.10 ± 8.56	0.31
RVSP, mmHg	26.96 ± 10.40(28.0)	27.31 ± 15.23(25.0)	0.85
Probability of PH (moderate or above)	18 (13.04)	22 (39.29)	0.000

LA_minor, minor dimensions of the left atrium; LA_major, major dimensions of the left atrium; LAA, area of the left atrium; IVSs, dimensions of the interventricular septum at end-systole; IVSd, dimensions of the interventricular septum at end-diastole; LVPWs, dimensions of the left ventricular posterior wall at end-systole; LVPWd, dimensions of the left ventricular posterior wall at end-diastole; LVEDV, left ventricular end-diastolic volume; LVESV, left ventricular end-systolic volume; LVEF_m mode, left ventricular ejection fraction was calculated by the Teichholz method; LVEF_simpson, LVEF calculated by bi-plane Simpson; E, early mitral filling velocity; A, late mitral filling velocity; EDT, E deceleration time; IVRT, isovolumetric relaxation time; RA_minor, the minor dimensions of the right atrium; RA_major, the major dimensions of the right atrium; RAA, area of the right atrium; RV_basal, the basal dimensions of the right ventricle; RV_mid, middle dimensions of the right ventricle; RV_long, longitudinal dimensions of the right ventricle; RV_proximal, the proximal dimensions of the right ventricular outflow tract; RV_distal, distal dimensions of the right ventricular outflow tract; RVESA, right ventricular end-systolic area; RVEDA, right ventricular end-diastolic area; RV_FAC, right ventricular systolic function was evaluated by the fractional area change; RVSP, right ventricular systolic pressure; PH, pulmonary hypertension.

### Electrocardiographic features in SSc and MCTD

A comparison of the frequency of PAC, PVC, RBBB, and RVH between the two groups is shown in Table 5. No statistical significance was achieved between the two groups.

**Table 5:** ECG findings in patients with scleroderma.

ECG finding	SSc (*N* = 138)	MCTD (*N* = 56)	*P-*value
AF, *n* (%)	2 (1.45)	1 (1.79)	0.99
PAC, *n* (%)	6 (4.35)	1 (1.79)	0.68
PVC, *n* (%)	3 (2.17)	0	–
Sinus tachycardia, *n* (%)	10 (7.25)	3 (5.36)	0.76
Sinus bradycardia, *n* (%)	6(4.35)	2 (3.57)	0.99
RBBB, *n* (%)	6(4.35)	6(10.71)	0.11
RVH, *n* (%)	4(2.90)	0	–
ST-T changes, *n* (%)	14 (10.14)	4 (7.14)	0.60

AF, atrial fibrillation; PAC, premature atrial contraction; PVC, premature ventricular contraction; RBBB, right bundle branch block; RVH, right ventricular hypertrophy.

## Discussion

To our knowledge, this is the first study to systematically analyze the differences in cardiopulmonary features between patients with SSc and MCTD using PFT, 6MWD, TTE, and 12-lead ECG. In this cross-sectional multi-center study, a similar incidence rate of ILD was observed between patients with SSc and MCTD. However, patients with SSc showed small airway dysfunction (SAD), manifesting as a declined FEF50 predicted value and a high percentage of FEF75 < 65% predicted value when compared to patients with MCTD. A previous study revealed that nearly half of the patients had been detected with SAD in patients with connective tissue disease-interstitial lung disease (CTD-ILD) (21). The role of SAD in the pathogenesis of ILD has not been well investigated. Pathological alterations in lung parenchyma impair small bronchial patency, while small bronchial obstruction triggers ventilation–perfusion mismatching and hypoxemia. Current expert consensus recommends PFT for screening and close monitoring of lung dysfunction in patients suspected of ILD (22). The results from the European Scleroderma Trials and Research (EUSTAR) registry showed the performance of PFT in predicting the occurrence of ILD, revealing that 9.7% of patients developed ILD for 24 months of follow-up, and DLCO < 80% measured at baseline moderately predicted ILD (23). Mittoo et al. suggested that the annual rate of decline in the predicted value of DLCO was −3.3 ± 7.7% in patients with SSc (24). Essentially, DLCO% was not only associated with the disease severity but also has the ability to anticipate organ damage and is closely linked with neoangiogenesis in nailfold videocapillaroscopy (25, 26).

Clinically, the study demonstrated that patients with MCTD had higher levels of TNT and BNP compared to patients with SSc. Furthermore, we found evidence that left ventricular systolic dysfunction was more severe in patients with MCTD compared to patients with SSc. Mean LVEDD, LVEDV, LVESV, and SV were higher and LVEF_simpson was lower in patients with MCTD than in those in the SSc group. In addition, two patients in MCTD had LVEF<50% vs. one patient in SSc. The above findings suggest the presence of myocardial injury or subclinical heart failure in the cohort. In this study, a higher proportion of patients with MCTD had anti-RNP positivity compared to those with SSc (42.86% vs. 12.32%, *p* < 0.000). Previous studies suggested that anti-RNP may stimulate the synthesis of IL-1 alpha and IL-6 in pulmonary artery endothelial cells and could be linked to microangiopathy (27). Additionally, the pathogenic effects of anti-RNP antibodies involve directly binding endothelial cell surfaces and further drive vascular endothelial dysfunction and vascular injury (28, 29). In some cases, ILD in MCTD patients is often accompanied by extensive pulmonary vascular disease, with patients showing enlarged pulmonary vessels and compromised pulmonary/aorta ratio, which further exacerbates the ventilation–perfusion imbalance (30).

Meanwhile, the right ventricular size, measured by RV_long, was larger, and a higher proportion of patients had with moderate-to-high probability of PH, as evaluated by echocardiography in the MCTD group. In patients with SSc, a solitary DLCO decline of ≤55% combined with a FVC/DLCO ratio of > 1.6 was found to be useful in identifying subsequent PAH (31). The diagnosis of PAH is confirmed by right heart catheterization, and its prevalence in MCTD may be similar or a little higher than that of SSc patients (32, 33). PAH in MCTD is usually secondary to intimal hyperplasia and medial hypertrophy of the pulmonary artery (34), which directly leads to increased right heart after-load and affects pulmonary function. The present study demonstrated that platelet counts were decreased in the MCTD group compared to the SSc group. Thrombocytopenia and positive anti-RNP were correlated with disease activity, disease progression, and an increased risk of PAH in immune-mediated inflammatory diseases (35–37). Furthermore, in patients with heart failure, moderate or severe thrombocytopenia was associated with higher all-cause mortality compared to low-degree thrombocytopenia or normal platelet counts (38, 39).

This multi-center study performed a comprehensive analysis of cardiopulmonary characteristics in patients with SSc and those with MCTD. However, limitations existed in our study. We did not include data on autoimmune treatment strategies and on the management of comorbidities in the study, which may limit our ability to fully exclude potential confounding effects of treatments on cardiopulmonary parameters. However, the comorbidity burden was comparable between the two groups, which may help alleviate the effect. Furthermore, patients in our study had a disease duration of 10 years on average, which could mean that the results cannot be extended to patients at an early stage, with active inflammation and severe cardiopulmonary compromise. Additionally, only a small number of patients with MCTD were included in the analysis because of the low prevalence of MCTD in Asia. There was difficulty in enrolling male patients with SSc or those with MCTD due to gender differences in the incidence of systemic sclerosis and mixed connective tissue disease, with a female-to-male ratio of approximately 3 ~ 4:1 or higher. Therefore, the results of the study may not be generalizable to male patients.

## Conclusion

We found a higher prevalence of small airway dysfunction in patients with SSc, while impaired left ventricular systolic function was more severe in patients with MCTD. These findings indicate that there was significant heterogeneity in cardiopulmonary involvement patterns, although these are both connective tissue diseases. Thus, differentiated follow-up strategies for cardiopulmonary function may be needed in patients with CTD, such as monitoring changes in pulmonary function, particularly FEF50 and FEF75, in patients with systemic sclerosis, while tracking variations in left ventricular function and the risk of pulmonary hypertension in the mixed connective tissue disease cohort via echocardiography.

## Data Availability

The original contributions presented in the study are included in the article/supplementary material; further inquiries can be directed to the corresponding authors.
